# Prevalence and determinants of workplace violence among nurses in public tertiary hospitals in Enugu state, Nigeria

**DOI:** 10.1371/journal.pone.0349187

**Published:** 2026-05-22

**Authors:** Nwanneka Chidinma Ghasi, Daniel Chukwuemeka Ogbuabor

**Affiliations:** 1 Department of Management, Faculty of Business Administration, University of Nigeria Enugu Campus, Enugu, Enugu State, Nigeriay of Management Sciences, University of Nigeria, Nigeria Enugu, Enugu State, Nigeria; 2 Department of Health Administration and Management, Faculty of Health Sciences and Technology, University of Nigeria Enugu Campus Enugu, Enugu State, Nigeria; University of Toronto, CANADA

## Abstract

**Background:**

Despite its impact on nurses’ well-being, hospital efficiency, and patients’ quality of care, published studies on workplace violence against nurses in sub-Saharan Africa are scarce. This study assessed the prevalence, characteristics, and determinants of workplace violence among nurses in public tertiary hospitals in Enugu State, Nigeria.

**Methods:**

The study was a cross-sectional survey among nurses in the four (4) public tertiary hospitals (n = 450) in Enugu State Nigeria. The nurses were selected from the hospitals using proportional stratified random sampling technique. Data were collected using a self-administered, workplace violence questionnaire for nurses. The outcome variable was nurses’ experiences of workplace violence in the past 12 months, scoring as yes or no. Workplace violence included verbal, physical, and sexual violence. Additionally, we collected socio-demographic and organizational characteristics of the nurses. Nurses’ socio-demographic factors, organizational characteristics and prevalence of workplace violence were summarized using descriptive statistics. We identified the predictors of workplace violence using bivariate analysis and binary regression. The significance level for all inferential analyses was p-value less than 0.05.

**Results:**

Overall, the prevalence of any workplace violence was 92.4%. The prevalence of verbal violence, physical violence, and sexual violence are 91.1%, 13.3%, and 2.0% correspondingly. Relatives of patients perpetrated most workplace violence among nurses in this study. Female nurses were more likely to experience verbal abuse than male nurses (COR = 2.84, 95%CI: 1.09–7.42, p = 0.002). Being a registered nurse (AOR = 2.06, 95%CI: 1.13–3.78, p = 0.019), direct contact with patients (AOR = 21.04, 95%CI: 3.17–139.82, p = 0.002), working in accident and emergency units (AOR = 6.91, 95%CI: 2.82–16.93, p < 0.001) increased the likelihood of physical violence against nurses. Direct contact with patients (AOR = 20.71, 95%CI: 3.54–121.12, p = 0.001) increased the likelihood of sexual violence.

**Conclusion:**

Workplace violence among nurses in public hospitals need to improve. Decision-makers and practitioners can incorporate these findings into safeguarding policies and strategies to improve nurses’ safety and monitor interventions to eliminate workplace violence against nurses in Nigeria.

## Introduction

Workplace violence (WPV) among nurses refers to incidents of mistreatment, intimidation, or assault of nurses while they are at work, which directly or indirectly threaten their safety, well-being, or health [1]. Due to their frontline roles, nurses are three times more likely to experience WPV than other health workers [2–5]. The prevalence of WPV among nurses varies widely across contexts [6–15]. Although patients and their relatives are the most prevalent people engaged in violent behaviours against nurses, coworkers, and other professional groups also perpetrate workplace violence against nurses [1,3,9,16–18]. Nonetheless, most nurses do report WPV due to lack of support, a lack of hospital reporting protocols, unclear reporting channels, increased workload, insufficient staffing, a poorly implemented visitor policy, and unfulfilled expectations [4,16,19–21]. Therefore, healthcare organizations need to enact occupational safeguarding legislation, enforce zero-tolerance policies, and adopt strategies to improve patient-nurse relationships and prevent WPV among nurses.

WPV contributes to the global nursing shortage of around 9 million and reduces the efficiency of healthcare organizations [2,22]. WPV reduces nurses’ work engagement and task performance [23], autonomy [22,24–26], and motivation and causes them to withdraw from co-workers and experience anxiety [10,16,27,28]. WPV predisposes nurses to burnout, long-term dissatisfaction, resentment, and the intention to leave their jobs [5,28,29]. WPV among nurses results in substance misuse, increased sick leave, absenteeism, reduced hospital effectiveness and efficiency, and career or death loss [2,3,10,19], and physical and mental injuries [23]. Additionally, WPV increases patients’ wait time and medication errors [22,24–26], and causes anger toward patients, a lack of empathy, and disengagement with patients [28,29].

Categories of WPV include verbal violence, physical violence, and sexual violence [8,30]. Verbal violence is an intentional use of devaluing or humiliating words, manners, or tone that significantly harms another person’s psychological integrity through coercion, threats, insulting, bullying, and emotional, psychological, and non-physical violence, causing harm to one’s mental well-being [6,9,12]. Globally, the prevalence of verbal violence ranged from 49.4% to 71.4% in Asian countries [20,21,25,31–33] 93.4% of nurses in Tunisia [11], 54% of European nurses [16], 52.6% to 94.3% in Middle-East countries [17,18,34]. In Nigeria, about 54.6% to 85.7% of nurses experienced verbal abuse, mainly in Outpatient Departments (OPD) and emergency units [3,12,27,35]. Determinants of verbal violence include insufficient lighting and a poor organizational culture [21], working in public hospitals and a lack of training on how to deal with workplace violence [33], nurses with work experience of 5–10 years compared to more junior nurses [20], and being a female nurses [6,9,10,27,29].

Physical violence refers to intentional acts to cause bodily harm to another individual, including beating, kicking, slapping, stabbing, shooting, pushing, biting, pinching, punching, hurling objects, strangling, dragging, pushing against walls, and threatening with weapons [9,12,17,32]. The prevalence of physical violence ranges from 9.1% to 78.30% among nurses in low-and-middle-income countries [5,17,18,20,21,23,31–33,36]. In African countries, the prevalence of physical violence against nurses were 6.7% to 42.9% [8–11,34]. In Nigeria, 12.6% to 34.3% of nurses experienced physical violence [19,27,35,37]. Determinants of physical violence include working in emergency rooms, outpatient clinics, or psychiatric units [21,33], inadequate skills to deal with workplace violence, working long hours and high workloads [33], working in acute and psychiatric ward [38], and direct contact with patients [1,39].

Sexual violence is a forceful sexual relation, threat of blackmail, offering gifts in exchange for sex, assault, and unwelcome behaviour that humiliates, threatens, or embarrasses the victim [9,18,32]. The prevalence of sexual violence among nurses ranged from 4.7 to 19.7% in Asia [5,23,31,32]. In Africa, the prevalence of sexual harassment ranges from 2.1% to 18.1% [8–10,34]. In Southwest Nigeria, the prevalence of sexual harassment among nurses was 6% in a general hospital in Osun state [12]. Direct patient contact is a risk factor for sexual violence [27]. Nurses who are appealing, amiable, and easygoing nurses are likelier to experience sexual harassment than other nurses [32].

Despite the growing literature on workplace violence, significant knowledge gap exists on the predictors of workplace violence against nurses in Nigeria [15,40]. Most studies in Nigeria have described the prevalence, patterns, causes, consequences, and factors associated with workplace violence broadly, often including nurses and other healthcare workers [3,12–14,27,35,37,41,42]. These studies were methodologically limited to test of association and did not include predictive analysis [3,12–14,27,35,37,41,42]. Furthermore, WPV studies involving nurses in tertiary hospitals were either limited to single centers or included lower-level hospitals [13,19,35,37,41,42]. To our knowledge, no research has investigated the predictors of verbal, physical, and sexual violence distinctively among nurses from many tertiary hospitals in Nigeria. The present study addresses this gap by examining the prevalence and determinants of the different dimensions of workplace violence among nurses in public tertiary hospitals in Enugu State, Nigeria. Such information will inform the development of context-specific policies to minimize violence and create a safe work environment for nurses in Nigeria.

## Methods and materials

### Study area

The study took place in Enugu metropolis, Enugu State, Nigeria. Enugu State is one of the five states in southeastern Nigeria and has four public tertiary hospitals: two teaching hospitals, one orthopedic hospital, and one neuropsychiatric hospital. The first teaching hospital is the University of Nigeria Teaching Hospital (UNTH), Ituku-Ozalla. Enugu State University Teaching Hospital (ESUTH), Parklane, Enugu, is the second academic hospital. The teaching hospitals serve as referral centers for specialized medical services, including surgeries, obstetrics and gynecology, pediatrics, and internal medicine. The orthopedic hospital is the National Orthopedic Hospital, Enugu (NOHE), a referral center for orthopedic and trauma cases in Nigeria’s South-East, South-South, and North-Central zones. The hospital is also a training center for orthopedic and trauma nurses, physiotherapists, occupational therapists, orthopedic technicians, and technologists. Federal Neuropsychiatric Hospital Enugu (FNHE) is a federal government-owned national mental health resource center for people with mental health problems. The neuropsychiatric hospital also serves as a regional center for treating mental health issues in South-East Nigeria.

### Study design and population

The study adopted a descriptive, cross-sectional survey research design. The study population includes all qualified nurses (n = 1435) working full-time in the four publicly owned tertiary hospitals at the time of the study. The study included all nurses with at least a registered nurse qualification who have worked in the hospital for at least one year and were willing and consented to participate. Nurses who were unwilling to participate, on leave at the time of data collection, or who had worked for less than one year in the hospitals were excluded from the study.

### Sample size determination and sampling technique

The minimum calculated sample size was 272. The study calculated the sample size using the workplace prevalence of 73.6% among nurses in a previous study, allowable error of 5%, and 10% non-response rate [42]. Nonetheless, the study sampled 450 nurses using a stratified proportionate sampling technique to select nurses in each hospital. The study allocated samples to each hospital in proportion to their nursing population: 157, 110, 42, and 141 to UNTH, NOHE, FNHE, and ESUTH, respectively.

### Data collection instrument

The instrument for data collection was a structured questionnaire. The questionnaire consisted of two sections. Section A addressed respondents’ socio-demographic and job-related characteristics, while Section B covered questions on prevalence, characteristics, prevention strategies, and reporting of workplace violence. The study adapted the workplace violence questions from workplace violence in the country case studies by the International Labour Organization, International Council of Nurses, World Health Organization, Public Services International [43]. The questionnaire investigated nurses’ experiences of any workplace violence in the past 12 months, scoring as yes or no. Each nurse was asked to respond to three questions on workplace violence experiences, including experience of verbal, physical, and sexual violence in the past 12 months. If a nurse experienced at least one or more of the WPV, the nurse is considered to have experienced WPV. Nurses who experienced any WPV were further asked to identify the primary perpetrator of the abuse and the main reason for the violence.

### Data collection method

The data collection took place between 13 November 2023 and 26 January 2024. The researcher and two research assistants conducted the data collection. The research assistants were biomedical science graduates. Prior to data collection, the researcher trained the research assistants on the tools, data collection procedure, and research ethics. The researcher and research assistants visited the hospitals and identified eligible nurses in collaboration with unit heads. In each hospital, eligible nurses received an information sheet, a consent form, and a questionnaire. After reading the information sheet, the nurse signed and returned the consent form to the data collector. The nurse then completed the questionnaire. The questionnaire took about 20 minutes to complete. Nurses, busy with clinical duties, took their time to complete and return the questionnaire the following day. A small proportion of nurses returned the filled questionnaires within two or three days. The collected data was kept confidential and used only for this study.

### Data analysis

The data collected was analyzed using Statistical Package for Social Sciences version 20. Nurses’ socio-demographic and organizational characteristics were summarized in frequencies and percentages using tables. The study also used descriptive statistics to present the prevalence of workplace violence. The study calculated a composite workplace violence defined as experiencing any of the three dimensions of workplace violence. We cross-tabulated workplace violence (any workplace violence, verbal, physical, and sexual violence) and socio-demographic and organizational factors to test for their association using Chi-square statistics.

Additionally, where the assumptions of Chi-square test were not met, particularly for sexual violence, the study used Fisher exact (two-tailed) tests. The significant variables in the bivariate analyses were analyzed using binary regression to test their relationship with workplace violence. The significance level for all inferential analyses was p-value less than 0.05.

### Ethical consideration

Ethical approval was obtained from the Research Ethics Committee of each of the four public hospitals in Enugu State (ESUT Teaching Hospital, Parklane-ESUTHP/C-MAC/RA/034/182; Federal Neuropsychiatric Hospital-FNHE/HTR/REA/VOL.11/092; National Orthopaedic Hospital-S.313/IV/2023/12/055; and University of Nigeria Teaching Hospital-UNTH/HREC/2023/11/799). The study ensured confidentiality and anonymity of participants. All respondents provided written informed consent.

## Results

### Socio-demographic characteristics of nurses

Almost 50% of the nurses in this study are over 40 years of age. Most nurses are female and married. About 68% of nurses have a Bachelor of Nursing degree. Approximately 58% of nurses have worked for more than 10 years as nurse (Table 1). About 9% of nurses in this study worked in the Neuropsychiatric hospital. About 50% of nurses work in special clinics and outpatient departments.

**Table 1:** Socio-demographic characteristics of nurses.

Sociodemographic factors		Frequency (n)	Percent (%)
Age group	21-30	79	17.6
	31-40	148	32.9
	41-50	186	41.3
	51 or more	37	8.2
Gender	Male	30	6.7
	Female	420	93.3
Marital Status	Married	360	80.0
	Never in Union	76	16.9
	Divorced or separated/widowed	14	3.1
Highest Qualification	RN/RM	143	31.8
	BSC/MSc	307	68.2
Years worked as Nurse	0-10	191	42.4
	11-20	201	44.7
	21 or more	58	12.9
Hospital Type	State Teaching Hospital	141	31.3
	Federal Teaching Hospital	157	34.9
	National Orthopaedic Hospital	110	24.4
	National Neuropsychiatric Hospital	42	9.3
Direct patient contact	No	8	1.8
	Yes	442	98.2
Current Unit	Inpatient ward	115	25.6
	OPD/Special Clinics	223	49.6
	Intensive Care Unit	34	7.6
	Theatre	28	6.2
	Accident & Emergency	50	11.1
Clients worked with	Children	123	27.3
	Adults	327	72.7
Number of nurses in unit	0-5	56	12.4
	6-10	125	27.8
	11-15	153	34.0
	16-20	116	25.8
Shifting work	Yes	428	95.1
	No	22	4.9

### Prevalence and characteristics of workplace violence among nurses

**Fig 1:**
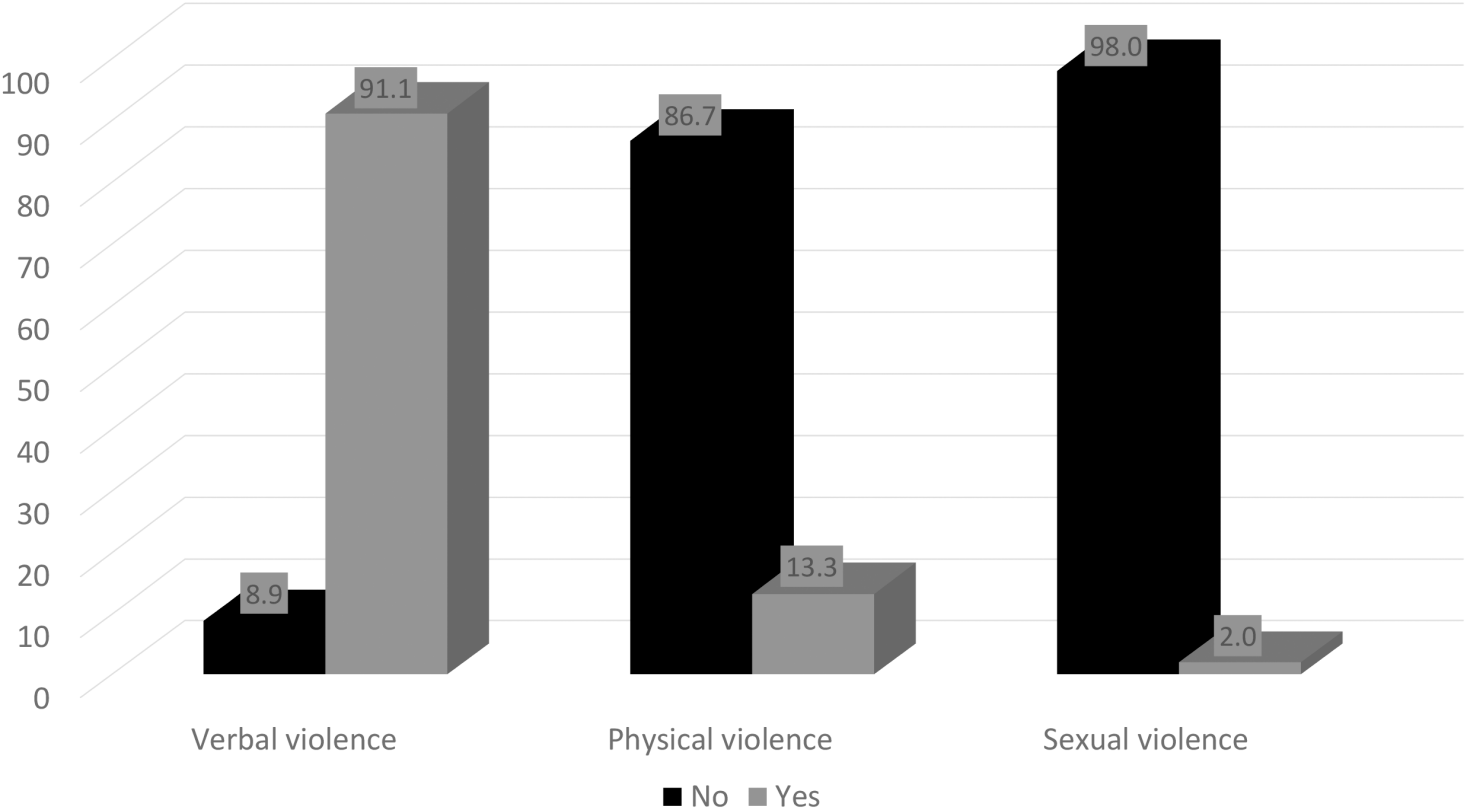
Prevalence of workplace violence among nurses in public tertiary hospitals in Enugu State.

Overall, 92.4% of nurses experienced at least one form of workplace violence in the 12 months preceding the study. The prevalence of verbal violence, physical violence, and sexual violence are 91.1%, 13.3%, and 2.0% correspondingly (Fig 1).

Relatives of patients accounted for 56% of verbal abuse, 78% of physical violence, and 33% of sexual violence among nurses in this study (Table 2). The leading reasons for verbal and physical violence were long waiting time, anxiety, unmet expectations, pain, and unavailability of needed services and medicine.

**Table 2:** Characteristics of workplace violence among nurses in public tertiary hospitals in Enugu State.

Parameters		Verbal violence		Physical violence		Sexual violence	
		Frequency (n)	Percent (%)	Frequency (n)	Percent (%)	Frequency (n)	Percent (%)
Primary abusive person	Patients	145	32.2	9	19.6	3	33.3
	Relatives of patients	252	56.0	36	78.3	3	33.3
	Nursing colleagues	20	4.4	1	2.2		
	Supervisors/other staff	2	.4			2	22.2
	Doctors	12	2.7			1	11.1
	Missing	19	4.2				
Main reason for violence	Long waiting time	85	18.9	12	21.1		
	Mental Patients	43	9.6	5	8.8		
	Anxiety/ fear/ stress	82	18.2	12	21.1		
	Influence of sickness/pain	59	13.1	7	12.3		
	influence of alcohol/drug	21	4.7	2	3.5		
	unmet expectation of patients/family	60	13.3	10	17.5		
	Staff attitude with patient	16	3.6	1	1.8		
	unavailability of needed service/medicine	56	12.4	1	1.8		
	lack of violence prevention methods	5	1.1	7	12.3		
	Information not provided	22	4.9				

### Factors associated with any workplace violence and its dimensions

No socio-demographic and organizational factor was significantly associated with workplace violence (Table 3).

**Table 3:** Socio-demographic and organizational factors associated with any workplace violence.

		No WPV n (%)	Yes, WPV n (%)	Chi-sq.	P-value
Age group	21-30	10(12.7)	69(87.3)	4.413	0.220
	31-40	10(6.8)	138(93.2)		
	41-50	13(7.0)	173(93.0)		
	51 or more	1(2.7)	36(97.3)		
Gender	Male	4(13.3)	26(86.7)	1.536	0.215
	Female	30(7.1)	390(92.9)		
Marital Status	Married	27(7.5)	333(92.5)	0.018	0.991
	Never in Union	6(7.9)	70(92.1)		
	Divorced or separated/widowed	1(7.1)	13(92.9)		
Highest Qualification	RN/RM	8(5.6)	135(94.4)	1.154	0.283
	BSC/MSc	26(8.5)	281(91.5)		
Years worked as Nurse	0-10	17(8.9)	174(91.1)	3.366	0.186
	11-20	16(8.0)	185(92.0)		
	21 or more	1(1.7)	57(98.3)		
Hospital Type	State Teaching Hospital	13(9.2)	128(90.8)	3.319	0.345
	Federal Teaching Hospital	7(4.5)	150(95.5)		
	National Orthopaedic Hospital	10(9.1)	100(90.9)		
	National Neuropsychiatric Hospital	4(9.5)	38(90.5)		
Direct patient contact	No	1(12.5)	7(87.5)	0.285	0.593
	YES	33(7.5)	409(92.5)		
Current Unit	Inpatient ward	12(10.4)	103(89.6)	6.952	0.138
	OPD/Special Clinics	17(7.6)	206(92.6)		
	Intensive Care Unit	4(11.8)	30(88.2)		
	Theatre	1(3.6)	27(96.4)		
	Accident & Emergency	0(0.0)	50(100.0)		
Clients worked with	Children	9(7.3)	114(92.7)	0.014	0.907
	Adults	25(7.6)	302(92.4)		
Number of nurses in unit	0-5	4(7.1)	52(92.9)	2.905	0.406
	6-10	10(8.0)	115(92.0)		
	11-15	15(9.8)	138(90.2)		
	16-20	5(4.3)	111(95.7)		
Shifting work	Yes	32(7.5)	396(92.5)	0.078	0.780
	No	2(9.1)	20(90.9)		

Gender was the only demographic and job-related factor significantly associated with verbal violence (p = 0.027). Marital status (p = 0.007), educational qualifications (p = 0.008), years worked as a nurse (p = 0.026), direct patient contact (p < 0.001), and current unit (p < 0.001) were significantly associated with physical violence (Table 4). Highest educational qualifications (p = 0.023), direct patient contact (p < 0.001), current unit (p = 0.023), and clients worked with (p = 0.002) had significant association with sexual violence (Table 4).

**Table 4:** Socio-demographic factors associated with any workplace violence.

		Verbal violence				Physical violence				Sexual violence			
		No	Yes	Chi-sq.		No	Yes	Chi-sq		No	Yes	Chi-sq.	
		n (%)	n (%)		P-value	n (%)	n (%)		P-value	n (%)	n (%)		P-value
Age group	21-30	10(12.7)	69(87.3)	1.68	0.641	68(86.1)	11(13.9)	0.27	0.996	75(94.9)	4(5.1)	5.01	0.171
	31-40	12(8.1)	136(91.9)			130(87.8)	18(12.2)			146(98.6)	2(1.4)		
	41-50	15(8.1)	171(91.9)			160(86.0)	26(14.0)			184(98.9)	2(1.1)		
	51 or more	3(8.1)	34(91.9)			32(86.5)	5(13.5)			36(97.3)	1(2.7)		
Gender	Male	6(20.0)	24(80.0)	4.90	0.027*	27(90.0)	3(10.0)	0.31	0.578	29(96.7)	1(3.3)	0.29	0.466
	Female	34(8.1)	386(91.9)			363(86.4)	57(13.6)			412(98.1)	8(1.9)		
Marital Status	Married	33(9.2)	327(90.8)	0.18	0.914	303(84.2)	57(15.8)	9.94	0.007**	351(97.5)	9(2.5)	2.3	0.317
	Never in Union	6(7.9)	70(92.1)			74(97.4)	2(2.6)			76(100.0)	0(0.0)		
	Divorced or separated/widowed					13(92.9)	1(7.1)			14(100.0)	0(0.0)		
Highest Qualification	RN/RM	11(7.7)	132(92.3)	0.37	0.543	115(80.4)	28(19.6)	7.08	0.008**	137(95.8)	6(4.2)	5.16	0.032*
	BSC/MSc	29(9.4)	278(90.6)			275(89.6)	32(10.4)			304(99.0)	3(1.0)		
Years worked as Nurse	0-10	20(10.5)	171(89.5)	1.63	0.444	157(82.2)	34(17.8)	7.26	0.026*	185(96.9)	6(3.1)	2.328a	0.312
	11-20	17(8,5)	184(91.5)			178(88.6)	23(11.4)			199(99.0)	2(1.0)		
	21 or more	3(5.2)	55(94.8)			55(94.8)	3(5.2)			57(98.3)	1(1.7)		
Hospital Type	EUSTH	15(10.6)	126(89.4)	6.403	0.094	117(83.0)	24(17.0)	7.39	0.061	136(96.5)	5(3.5)	6.58	0.087
	UNTH	7(4.5)	150(95.5)			145(92.4)	12(7.6)			157(100.0)	0(0.0)		
	NOHE	12(10.9)	98(89.1)			94(85.5)	16(14.5)			108(98.2)	2(1.8)		
	FNHE	6(14.3)	36(85.7)			34(81.0)	8(19.0)			40(95.2)	2(4.8)		
Direct patient contact	No	1(12.5)	7(87.5)	0.131	0.717	3(37.5)	5(62.5)	17.04	<0.001***	6(75.0)	2(25.0)	21.98	0.009**
	Yes	39(8.8)	403(91.2)			387(87.6)	55(12.4)			435(98.4)	7(1.6)		
Current Unit	Inpatient ward	14(12.2)	101(87.8)	4.37	0.358	105(91.3)	10(8.7)	30.87	<0.001***	112(97.4)	3(2.6)	11.39	0.023*
	OPD/Special Clinics	19(8.5)	204(91.5)			201(90.1)	22(9.9)			222(99.6)	1(4)		
	Intensive Care Unit	4(11.8)	30(88.2)			29(85.3)	5(14.7)			31(91.2)	3(8.8)		
	Theatre	1(3.6)	27(96.4)			24(85.7)	4(14.3)			27(96.4)	1(3.6)		
	Accident & Emergency	2(4.0)	48(96.0)			31(62.0)	19(38.0)			49(98.0)	1(2.0)		
Clients worked with	Children	11(8.9)	112(91.1)	0.001	0.980	106(86.2)	17(13.8)	0.035	0.852	118(95.9)	5(4.1)	3.683	0.067
	Adults	29(8.9)	298(91.1)			284(86.9)	43(13.1)			323(98.8)	4(1.2)		
Number of nurses in unit	0-5	4(7.1)	52(92.9)	6.52	0.089	51(91.1)	5(8.9)	2.34	0.505	54(96.4)	2(3.6)	0.85	0.837
	6-10	11(8.8)	114(91.2)			111(88.8)	14(11.2)			123((8.4)	2(1.6)		
	11-15	20(13.1)	133(86.9)			129(84.3)	24(15.7)			150(98.0)	3(2.0)		
	16-20	5(4.3)	111(95.7)			99(85.3)	17(14.7)			114(98.3)	2(1.7)		
Shifting work	Yes	38(8.9)	390(91.1)	0.001	0.973	368(86.0)	60(14)	3.56	0.059	420(98.1)	8(1.9)	0.77	0.366
	No	2(9.1)	20(90.9)			22(100)	0(0.0)			21(95.5)	1(4.5)		

*, **, ***Significance at p-value <0.05; <0.01 and <0.001, respectively.

### Predictors of verbal, physical, and sexual violence

The predictors of verbal, physical and sexual violence are shown in Table 5. We reported the unadjusted, crude odd ratio (COR) for gender, since gender was the only demographic and job-related factor associated with verbal violence included in binary regression for verbal violence. Female nurses were more likely to experience verbal abuse than male nurses (COR = 2.84, 95%CI: 1.09–7.42, p = 0.002).

**Table 5:** Predictor of verbal violence among nurses in public hospitals in Enugu State.

Explanatory variable		Verbal violence		Physical violence		Sexual violence	
		COR	Sig.	AOR	Sig.	AOR	Sig.
Gender	Female	2.84 (1.09-7.42)	0.033*				
	Male	1.00					
Qualification	RN/RM			2.06 (1.13-3.78)	0.019*		
	BSC or more			1.00			
Direct patient contact	Yes			21.04(3.17-139.82)	0.002**	20.71 (3.54-121.12)	0.001**
	No			1.00		1.00	
Current work unit	OPD/Special Clinics			1.27 (0.57-2.86)	0.561		
	Intensive Care Unit			1.83 (0.56-5.94)	0.314		
	Theatre			0.47 (0.08-2.68)	0.394		
	Accident & Emergency			6.91 (2.82-16.93)	<0.001***		
	Inpatient ward			1.00			
	Constant	4.00	0.002	0.07	<0.001	0.02	<0.001

*, **, ***Significance at p-value <0.05; <0.01 and <0.001, respectively; COR = Odd ratio; AOR = Adjusted odd ratio.

Although marital status, highest qualification, years worked as a nurse, direct patient contact, and current unit had significant association with physical violence, marital status and years worked as a nurse were not significant in the unadjusted binary regression. Being a registered nurse (AOR = 2.06, 95%CI: 1.13–3.78, p = 0.019), direct contact with patients (AOR = 21.04, 95%CI: 3.17–139.82, p = 0.002), working in accident and emergency units (AOR = 6.91, 95%CI: 2.82–16.93, p < 0.001) increased the likelihood of physical violence against nurses.

Regarding sexual violence, highest qualification, direct patient contact, and current unit were included in the binary regression, but only direct patient contact remained significant in the unadjusted model. Direct contact with patients (AOR = 20.71, 95%CI: 3.54–121.12, p = 0.001) increased the likelihood of sexual violence.

## Discussion

The study found a high prevalence of workplace violence in public tertiary hospitals in Enugu metropolis, which is higher than results from previous Nigerian and African studies [7–13,27,35,37,42]. One reason for this variation is that some previous studies included nurses in secondary health care and private health facilities, unlike the current study, which focused on nurses in publicly owned tertiary hospitals. It might also be that nurses in secondary and private hospitals underreported their experiences of workplace violence, given that WPV is an unpleasant part of the nursing profession, but reporting is unimportant [12,20]. Underreporting accounts for the low prevalence in Ethiopia [9]. Consistent with prior evidence [12,17], verbal violence is the most significant driver of the high prevalence of WPV in the current study

The high prevalence of verbal violence found in the current study is consistent with evidence from other countries [11,17,18,20,33,36]. Nevertheless, other studies found a lower prevalence of verbal violence [8,12,16,21,25,31,32,34]. The mixed findings on verbal violence across different settings indicate a need for evidence-informed, context-specific responses to reduce verbal violence. In the current study, patients’ relatives and patients were the most significant people perpetrating verbal violence against nurses. Prior studies also found that the perpetrators of verbal abuse are primarily patients and their relatives [9,10,18,20,35]. In our study, the critical reasons for verbal violence were long waiting times, unmet expectations of patients, influence of sickness or pain, and unavailability of needed services/medicines. Addressing these causes of WPV would be a meaningful change to reduce verbal violence among nurses in tertiary hospitals.

The study found that female nurses were likelier to experience verbal violence than male nurses in this study. This finding is consistent with the evidence from existing studies indicating that violence against nurses is more common among female nurses [6,9,10,27,29]. One possible cause of verbal violence against female nurses is the higher proportion of female nurses compared with male nurses in the nursing profession, given that the nursing profession is more accessible to women [29]. Another reason might be the negative community attitude toward female power and ability, where males are superior to women, and women are often subject to derogatory comments [9,10].

The prevalence of physical violence in the current study is consistent with evidence from previous studies [9,19,31,35,37,39]. While our prevalence is lower than the findings from other studies [5,11,16,17,27,32,33,36], the present study found higher a prevalence than evidence from Rwanda [8]. The variations in prevalence result from methodological differences: sampling exclusively mental health or emergency nurses and a mix of public and private nurses. Also, underreporting physical violence varies across settings depending on the availability of WPV prevention policies and reporting systems in the hospitals. Because of lower rates of reports, infrequent incidents of physical violence, which serve as warning signs of future physical violence, are tolerated [6,29]. The most frequently abusive persons are patients’ relatives. Long waiting times, anxiety, unmet expectations of patients and families, and the influence of pain were the leading reasons for physical violence. Evidence shows that many patients daily may overwhelm hospital staff, leading to long waiting periods and making patients and their relatives irritable [39].

The finding that nurses with lower qualifications in this study had increased odds of physical violence is consistent with the evidence that lower nursing qualifications increased the odds of physical violence among nurses [39]. In contrast, our findings differ from other preceding studies indicating that nurses were more likely to experience WPV if they held bachelor’s degrees or higher levels of education [17,32,36]. Evidence in Japan also shows that licensed nurses experienced more WPV than assistant nurses because of their role in convincing patients to take medicine or medical treatments or administer forced treatments [38]. Nurses with lower qualifications lack the experience, maturity, and ability to manage potentially violent circumstances or patients [39].

Consistent with previous studies [1,39], having direct contact with patients increased the likelihood of physical violence among nurses in public tertiary hospitals in the present study. This finding should be cautiously interpreted given the wide confidence interval of the odd ratio. In clinical settings, nurses’ work usually brings them close to the patients and their relatives, which puts them at a higher risk of being abused physically than other health professionals when patients or their relatives perceive a delay in accessing healthcare or when care expectations are not [39]. Nurses’ position exposes them to violence related to the environment as the nurses who are usually in direct contact with the patient were more apt to violence than the nursing supervisor or the nurses working in offices [1].

This study’s finding that nurses working in the accident and emergency units were more likely to experience physical violence is similar to those found by previous studies [8,35,39]. The interdependence theory of aggression explains the pathway to violence in emergency units. Interaction between patients and nurses depends on the patient’s factors, the nurse’s approach towards them, and how patients and nurses deal with stress. The accident and emergency units care for many severely ill patients who need nursing care and regular monitoring. For example, communicating poor prognoses to the patients and patient’s relatives may create unnecessary anger and anxiety, leading to violence against the nurses. Our finding highlights a need for hospital decision-makers to direct a violence prevention program to high-risk areas, such as emergency units, with patients whose behaviour can be affected by health conditions and drugs [21].

We found a considerably low prevalence of sexual violence among nurses in this study, which is consistent with evidence from Rwanda [8]. Nonetheless, our finding is low compared to studies conducted in China, Ethiopia, and Indonesia [5,9,10,31]. Sexual violence is stigmatizing and may be the reason for underreporting sexual violence in our study. The findings of this study further show that having direct contact with patients increased the likelihood of sexual violence among nurses in public tertiary hospitals in Enugu State. This finding is consistent with a previous study, which revealed that most of the nurses who experienced sexual harassment had direct patient contact [27]. However, this finding has a wide confidence interval, indicating substantial imprecision, and should be interpreted with caution.

Our study fills an important knowledge gap in literature by providing evidence of the social and organizational predictors of individual dimensions of workplace violence in public tertiary hospitals in South-East, Nigeria. Nonetheless, the findings may not be generalizable to the other geopolitical zones of Nigeria or nurses in private health facilities. Also, as with cross-sectional surveys, our study does not establish any causal inferences. Future studies may consider conducting longitudinal studies, where data is collected from nurses over time. This would allow the researchers to observe changes, understand the temporal sequence of events, and assess potential causal relationships more confidently. Moreover, the study’s findings could suffer from social desirability bias, where nurses may have presented answers that are more socially acceptable than their actual experiences, leading to the underreporting of physical and sexual violence and the over-reporting of verbal violence.

## Conclusion

The study provided evidence on the prevalence and determinants of workplace violence. The burden of workplace violence among nurses is high, especially verbal and physical violence. Gender differences were significant in verbal violence. Nurses’ qualifications, direct patient contact and working in accident and emergency units significantly increase the risk of physical violence. Direct contact with patients increases the risk of sexual violence among nurses. Health policymakers, practitioners, and health facility managers need to incorporate these findings into safeguarding policies and strategies to improve nurses’ safety, intervene promptly in cases of workplace violence, and monitor interventions to eliminate workplace violence against nurses in Nigeria.

## Abbreviations

ESUTH: Enugu State University Teaching Hospital
FNHE: Federal Neuropsychiatric Hospital Enugu
NOHE: National Orthopaedic Hospital Enugu
OPD: Outpatient department
UNTH: University of Nigeria Teaching Hospital
WPV: Workplace violence
